# AVD Best Cited Articles 2021

**DOI:** 10.3400/avd.bca.21-01000

**Published:** 2021-12-25

It is with a great pleasure to announce the top three most-cited AVD articles on Web of Science.

## No. 1

**Review Article**

**Visceral Artery Aneurysms and Pseudoaneurysms? Should They All be Managed by Endovascular Techniques?**

Alfredo C. Cordova and Bauer E. Sumpio

**Citation: 59**

Published: 2013 (Vol. 6 No. 4: 687-693)

DOI: 10.3400/avd.ra.13-00045

## No. 2

**Review Article**

**Compression Therapy: Clinical and Experimental Evidence**

Hugo Partsch

**Citation: 56**

Published: 2012 (Vol. 5 No. 4: 416-422)

DOI: 10.3400/avd.ra.12.00068

## No. 3

**Review Article**

**The Relationship between Vascular Function and the Autonomic Nervous System**

Eisuke Amiya, Masafumi Watanabe, and Issei Komuro

**Citation: 46**

Published: 2014 (Vol. 7 No. 2: 109-119)

DOI: 10.3400/avd.ra.14-00048

## Notes

The ranks are based on the citation counts on Web of Science, as of November 10, 2021.

